# Substance Use, Health, and Adverse Life Events amongst Amphetamine-Type Stimulant Users in North East England: A Cross-Sectional Study

**DOI:** 10.3390/ijerph19126996

**Published:** 2022-06-07

**Authors:** Emma Audrey Adams, Liam Spencer, Michelle Addison, William McGovern, Hayley Alderson, Mark Adley, Ruth McGovern, Eilish Gilvarry, Eileen Kaner, Amy O’Donnell

**Affiliations:** 1 Population Health Sciences Institute, Newcastle University, Newcastle upon Tyne NE3 4ES, UK; emma.adams@newcastle.ac.uk (E.A.A.); liam.spencer1@newcastle.ac.uk (L.S.); hayley.alderson@newcastle.ac.uk (H.A.); m.adley2@newcastle.ac.uk (M.A.); r.mcgovern@newcastle.ac.uk (R.M.); eilish.gilvarry@cntw.nhs.uk (E.G.); eileen.kaner@newcastle.ac.uk (E.K.); 2 Department of Sociology, Durham University, Durham DH1 3HN, UK; michelle.addison@durham.ac.uk; 3 Department of Social Work, Education and Community Studies, Northumbria University, Newcastle upon Tyne NE7 7XA, UK; william.mcgovern@northumbria.ac.uk

**Keywords:** cross-sectional survey, amphetamine, drug misuse

## Abstract

Amphetamine-type stimulants (ATS) are the second most commonly used class of illicit drugs globally, yet there is limited understanding of which factors contribute to different pathways of ATS use. We sought to compare current, former, and exposed non-ATS users’ substance use, mental/physical health, and adverse life experiences. A cross-sectional survey, using computer-assisted personal interview software, was conducted between June 2018 and March 2019 in North East England. Quota-based sampling was used to recruit 389 individuals (aged 18 to 68; 52.6% male): 137 current ATS users; 174 former users; and 78 exposed non-users. Standardized screening questionnaires captured current/prior substance use. Participants self-reported diagnoses of selected physical and mental health disorders and specific adverse life experiences. Analysis used descriptive statistics and comparative tests (including chi-square, Kruskal–Wallis and Mann–Whitney U). Early exposure to illicit substances, challenging mental health, and certain adverse life experiences (such as growing up in statutory care) were more common in individuals currently using ATS compared to those who had never used or stopped using stimulants. Multi-level interventions are needed that address the mental health, social, and economic needs of people with dependent drug use. These could include targeted efforts to support children growing up under care, integrated mental health and substance use support, and joined-up substance use interventions reflective of wider structural factors.

## 1. Introduction

Amphetamine-type stimulants (ATS) cover a group of drugs including amphetamine, methamphetamine, and 3,4-Methylenedioxymethamphetamine (MDMA), and after cannabis, they are the second most commonly used class of illicit drugs globally [1]. Between 2008 and 2017, global ATS police seizures increased fourfold [2], with a marked rise in new/novel psychoactive substances (NPS) [2,3]. Despite declining use in recent years, in England and Wales, around 9% of adults aged 16 to 59 reported ever using amphetamines [4]. ATS can lead to a range of individual harms, including dependency, premature death, physical and mental ill-health, and impaired social functioning [5,6,7,8,9]. However, the burden of ATS consumption, particularly at problematic levels, extends beyond the individual to wider social harms such as lost productivity, environmental damage, disruption of family life, and various crime-related activities [6,10,11]. Currently, there is no effective standardised pharmacotherapy for problematic ATS use [12]; the primary interventions are psychosocial therapies [13,14]. A recent network meta-analysis suggested combined a community management plus a community reinforcement approach are the most effective and acceptable interventions for problematic ATS use [15].

People start and continue to use ATS for different reasons. Qualitative evidence has highlighted the diversity of ATS users and the varied individual, social, and environmental factors that shape stimulant consumption at different points in their lives [8,16,17]. Several studies have identified an association between experiencing adverse childhood events (ACEs), such as parental substance use, physical or sexual abuse, parental incarceration, and subsequent ATS use, particularly methamphetamine use [18,19,20]. Adverse events experienced in adulthood such as unemployment, homelessness, sexual violence, and loss of a relationship have also been associated with ATS consumption [16,17,21,22,23]. However, there is limited quantitative evidence assessing how these factors and previous life events influence and shape substance use behaviour in different profiles of ATS users.

The wider ATTUNE study aimed to understand which factors shape ATS use pathways across five European countries (Czech Republic, Germany, the Netherlands, Poland, and England) using a sequential, exploratory mixed-methods design spanning three phases (described in full in the published study protocol [24]). First, a systematic review and synthesis of international qualitative literature highlighted the heterogeneity of ATS users and the complex interplay of individual, social, and environmental factors shaping ATS initiation and trajectories [17]. Second, informed by the review findings, qualitative interviews with ATS users and exposed non-users explored perceived critical turning points into and out of stimulant use, with English data suggesting that mental and physical health, and adverse life events influenced drug use pathways [8]. Cross-European analysis of life chart data captured during these interviews also found that the number and type of negative life events was significantly associated with drug trajectories [16]. Third, a cross-sectional computer-assisted personal interview (CAPI) survey of current, former, and exposed non-ATS users was conducted to validate and enhance the generalisability of the interview findings [24].

In this paper, we report the English findings from this final phase of the ATTUNE study, which sought to describe patterns in participants’ use of licit and illicit substances, including age of initiation; self-rated mental and physical health; and exposure to adverse life experiences and trauma in childhood and adulthood.

## 2. Materials and Methods

### 2.1. Design and Population

Using a cross-sectional sample, a CAPI survey was conducted in North East England between June 2018 and March 2019.

Three different groups of adults (18+) were targeted for recruitment: (1) current users (defined as having used ATS at least once within the last three months); (2) former users (defined as having previously used ATS but not within the previous year); and (3) exposed non-users (defined as never having used ATS despite access and opportunity to do so, such as in social settings and through friends and acquaintances). Embedded within the overall European study sample [24], non-probability quota-based sampling was used to recruit a target sample of 375 individuals for the English survey, comprised of 150 current ATS users, 150 former ATS users, and 75 exposed non-ATS users. Working with health, social care, and third-sector providers, participants were recruited via a range of means to maximise variation in experience and boost representativeness in our sample. The study was advertised through multiple networks, local buildings, and social media and employed snowballing techniques.

### 2.2. Procedures

The survey was administered face-to-face by a researcher using the CAPI method via tablet-computer in a variety of settings (e.g., local service providers, universities, and public places). All survey administrators were experienced in either conducting qualitative interviews or administering quantitative surveys with substance users. Prior to undertaking any data collection, a training session took place to introduce researchers to the study and to give them an opportunity to review and pilot survey questions and associated materials. Individuals needed sufficient verbal and cognitive skills (such as good level of English language, had capacity to consent, not under the influence of drugs or alcohol) to be able to participate in a survey, and this was confirmed through gatekeepers in practice. Where necessary, researchers supported participants to complete the survey through clarifying and reading out questions. Potential participants were allocated to the appropriate user group (current, former, or exposed non-ATS user) via an electronic screening tool. Surveys took approximately 60 min to complete depending on participant (i.e., complexity of substance use profile). Participants were given a GBP 10 voucher remuneration.

### 2.3. Ethical Approval

Ethical approval for the study was granted by North East-Newcastle and North Tyneside 2 Research Ethics Committee (REC reference:17/NE/0283) on 25 May 2018. All participants provided informed consent prior to data collection commencing.

### 2.4. Measures

Survey questions captured socio-demographic characteristics, including age, gender, ethnicity, relationship status, annual net income, employment status, highest completed level of education, and criminal justice involvement.

Standardized and validated screening tools and questionnaires were used to assess current and prior substance use. Three questions from the Alcohol Use Disorders Identification Test-Consumption version (AUDIT-C) [25] were used to assess the level of risk due to alcohol consumption using a Likert scale ranging from 0 to 4 (total scores range 0–12). A summative score of 5 or more suggests ‘hazardous’ (defined as drinking at a level that places an individual at risk of negative health events) or ‘harmful’ (defined as drinking at a level that results in adverse events) consumption [26]. Tobacco use was collected categorically with response options of never smoked, current smoker, or former smoker. Originally developed to measure heroin use, the Severity of Dependence Scale (SDS), a five-item scale with scores ranging from 0 to 15, was administered to current and former ATS users to provide an indication of the severity of any substance use including co-occurring opioid use [27,28]. Based on existing evidence, scores >4 indicated problematic ATS use [28]. SDS questions required participants to consider the 12 months they used ATS most often and were asked about their ATS use during this time, for example: “did you ever think your use of ATS was out of control? Did the prospect of missing a shot/snort (or dose) ever make you anxious or worried.” Any previous experience of injecting drug use, contact with specialist treatment services, and lifetime prevalence (ever used) ATS and/or non-stimulant type illicit substances were collected using binary categories. Age of initiation of substance use was collected as a continuous variable.

Additional questions were based on the key themes emerging from prior qualitative phases of the ATTUNE study [8,9]. Self-reported diagnoses of selected mental disorders were collected, and participants rated their physical and mental health on a scale of one (‘very bad’) to ten (‘excellent), although these diagnoses were not corroborated by medical records. Experiences of adverse life experiences in childhood (aged 16 and under) and adulthood (aged over 16) were collected as binary categories.

### 2.5. Analysis

Descriptive statistics were used to compare the three groups in terms of: socio-demographic characteristics; rates of licit/illicit substance use; mental and physical health; and adverse life experiences (childhood and adulthood).

Counts (percentages) or means (standard deviations) were calculated for each variable as appropriate by study group and for the entire sample. Differences between rates of nominal categorical data were compared using the Chi-square test. Differences between means of continuous and ordinal categorical data were compared using the Kruskal–Wallis test or Mann–Whitney U, with post-hoc Dunn–Bonferroni pairwise tests conducted to detect significant differences between study groups. Significance levels were set at *p* ≤ 0.05. All analysis was conducted using SPSS Software Version 26.

## 3. Results

### 3.1. Sample Characteristics

A total of 389 individuals contributed data between June 2018 and March 2019, comprised of current ATS users (*n* = 137), former ATS users (*n* = 174), and exposed non-ATS users (*n* = 78). Participants were drawn from across North East England. However, there was a high concentration of survey respondents from urban areas in the region, including Newcastle upon Tyne and Darlington.

Table 1 presents the demographic information of all survey participants. Compared to exposed non-users, current/former ATS users were more likely to earn less than GBP 18 k (net) (90.4% and 85.1% compared to 59.0%, *p* < 0.001), have a basic level of education (40.1% and 32.8% compared to 9.0%, *p* < 0.001), and to have been in prison (51.1% and 36.2% compared to 6.4% for exposed non-users, *p* < 0.001). They were also less likely to be employed (10.2% and 22.4% compared to 53.8%, *p* < 0.001). Post-hoc tests for age showed significant difference between current and exposed non-ATS users (*p* = 0.039).

### 3.2. Licit and Illicit Substance Use

Most participants in all groups drank alcohol, with 54.0% of all participants scoring 5 or more on the AUDIT-C questionnaire, suggesting hazardous drinking levels. The mean AUDIT-C score did not differ significantly by study group (H(2) = 0.017, *p* = 0.992). Smoking rates varied, with higher levels of current tobacco use found in current and former ATS users (85.4% and 77.0%, respectively) compared to exposed non-users (30.8%, *p* < 0.001). Table 2 provides full details of participants’ use of licit and illicit substances.

The age at which first use of, or exposure to, any ATS occurred varied significantly between groups (*p* < 0.001). Post-hoc tests for age of first use or exposure showed significant differences between current and former ATS users (*p* < 0.001), current and exposed non-ATS users (*p* < 0.001), and former and exposed non-ATS users (*p* = 0.34). Current ATS users reported having first used/been exposed to any ATS at an earlier age compared to other groups (16 years, compared to 18 years for former users, and 21 years for exposed non-users; U = 7695.000, *p* = 0.001).

According to the SDS, current users were more likely to score positive (i.e., report potential psychologically dependent levels of any substance use) compared to former users (U = 9066.500, *p* < 0.001). Current ATS users also reported significantly higher rates of previous injecting drug use (42.3%) compared to former users (17.2%), with no exposed non-users ever having injected drugs (*p* < 0.001). Current and former ATS users also reported higher rates of previous contact with specialist drug treatment services compared to exposed non-ATS users (76.2% and 63.8% compared to 17.9%, *p* < 0.001). Post-hoc tests for SDS showed a significant difference between current and former ATS users (U = 9066.500, *p* < 0.001). See Table 2 above for full details of participants’ any illicit substance use.

The types of ATS consumed varied by participant group, with current ATS users more likely to have ever used methamphetamine and stimulant-type NPS compared to those no longer using ATS (24.8% compared to 10.9%, respectively, for methamphetamine (*p* = 0.001); 50.4% compared to 25.3%, respectively, for stimulant type NPS). There were no significant differences between groups found for other types of ATS (such as MDMA and amphetamine medicine). Post-hoc tests for age of first use for amphetamine in years showed a significant difference between current and former ATS users (U = 7695.000, *p* = 0.001). Post-hoc tests for age of first use for MDMA showed a significant difference between current and former ATS users (U = 7582.500, *p* = 0.013). Table 3 provides further details on ATS and other illicit drugs.

All participants were asked whether they had ever used other (non-ATS) illicit substances, including cannabis, cocaine, and hallucinogens, and their age at first use (in years). Whilst most participants had used cannabis, rates were significantly higher in current and former ATS users compared to exposed non-ATS users (*p* < 0.001). Current users reporting an earlier mean age of first use of cannabis (14 years) compared to either former ATS users (16 years) or exposed non-ATS users (19 years) (*p* < 0.001). Post-hoc tests showed significant differences for age of first use of cannabis between current and former ATS users (*p* < 0.018); current and exposed non-ATS users (*p* < 0.001); and former and exposed non-ATS users (*p* < 0.001). Current or former ATS users were significantly more likely to have also used opioids compared to exposed non-ATS users (*p* < 0.001), although the age of initiation (in years) did not differ significantly (*p* = 0.309). Rates of use of cocaine/crack and hallucinogens varied between groups (both *p* < 0.001). However, the age of first use only differed significantly for cocaine/crack (*p* = 0.01). Post-hoc Dunn’s pairwise tests were carried out for the three groups and showed that current ATS users first used cocaine/crack at a significantly younger age than exposed non-ATS users (21 compared to 28 years, *p* = 0.011). Former ATS users first used cocaine/crack at a borderline significantly younger age than exposed non-ATS users (22 compared to 28 years, *p* = 0.049).

### 3.3. Mental and Physical Health

Depression, psychosis, and borderline personality disorder were the most frequently self-reported mental disorders amongst all participants. Table 4 presents additional information on self-reported mental health disorders and current physical and mental health. Most respondents in all three groups reported having experienced depression at some point during their lifetime (*n* = 281, 72.8%). However, rates were highest amongst current and former ATS users: 76.1% and 75.3%, respectively, compared to 61.5% in exposed non-users, with the difference between groups being significant (*p* = 0.043). Current ATS users reported the highest lifetime rates of psychosis (31.3% compared to 14.9% for former ATS users and 3.8% for exposed non-users, *p* < 0.001). Differences between groups for prevalence of self-reported ADHD (Attention Deficit/Hyperactivity Disorder), borderline personality disorder, and eating disorder were not significant. Post-hoc tests for physical health showed significant differences between current and exposed non-ATS users (*p* < 0.001) and former and exposed non-ATS users (*p* < 0.001). Post-hoc tests for mental health showed significant differences between current and exposed non-ATS users (*p* < 0.001) and former and exposed non-ATS users (*p* = 0.001).

Exposed non-ATS users rated both their physical and mental health significantly higher than current or former ATS users, with current ATS users rating their physical and mental health as the lowest out of all three groups (both *p* < 0.001).

### 3.4. Specific Adverse Life Events and Experiences before and after Age 16

Before the age of 16, around 1 in 10 participants reported experiencing sexual abuse/assault and/or the death of a parent (9.1% and 10.9% respectively), with no significant differences between user groups (*p* = 0.888 and *p* = 0.808). Whilst a higher proportion of current and former users reported having experienced parental substance use (29.6% and 24.9%, respectively) compared to those who had never used ATS (16.7%), this difference was not significant (*p* = 0.108). Only rates of participants who reported having grown up in care (defined as statutory or state-provided care for children where parental support is absent or deemed unsafe) differed significantly between groups (*p* = 0.002), with current users more likely to report this experience (23%) compared to either former users or those who had never used ATS (11% and 7.7%, respectively). See Table 5 for further details.

After 16 years, a higher proportion of current and former ATS users reported having experienced sexual assault (18.5% and 23.1%, respectively) compared to exposed non-ATS users (10.3%); although these differences were not statistically significant (*p* = 0.054). However, there were significant differences between groups in relation to measures of experiencing adverse living circumstances as an adult: 64.4% and 50.9% of current and former ATS users had experienced homelessness, compared to 15.4% of those who had never used ATS (*p* < 0.001). Moreover, 43.7% and 36.4% of current and former ATS users had experienced breakdown in living arrangements within parental/family home, compared to 16.7% of those who had never used ATS, and again, this difference was significant (*p* < 0.001).

## 4. Discussion

Most participants in all three groups had previously used licit substances (alcohol and tobacco) and cannabis, but significant differences emerged in their exposure to or use of ATS and other illicit substances. In particular, people currently using ATS reported having first used amphetamines and MDMA at a significantly earlier age, were more likely to have previously injected substances, and score positive for substance use dependency compared to either people who formerly used ATS or exposed non-users. Current ATS use was also associated with higher rates of lifetime use of other illicit substances, and earlier age of initiation of cannabis use.

These findings corroborate those of other international studies that have explored the substance use profile of ATS users, showing high rates of other substance or polysubstance use prior to ATS initiation, both licit and illicit [18,29]. Existing literature also suggests that the early initiation of substance use is a predictor of subsequent dependence [29,30,31], with early initiation of amphetamine use specifically, associated with higher dependence and severity of use in later life and increased risk for a range of mental health, substance use, and psychosocial problems in young adulthood [7,29,30,31,32]. In contrast, people who were exposed to ATS later in life (after 20 years of age) were more likely not to initiate stimulant use. As such, our findings confirm the need to prevent and/or delay early initiation of ATS to reduce later life dependency and associated adverse outcomes.

We also found evidence of significantly higher levels of mental and physical ill-health amongst current ATS users compared to the other groups [8,9]. In particular, current ATS users self-reported higher rates of psychosis and depression compared to other participant groups and self-rated their mental and physical health as poorer. Other data confirm higher rates of depression diagnoses in the North East compared to England as a whole (10% to over 17% compared to 9.1% for England [33]); our findings suggest ATS users are particularly likely to experience poor mental health, with over 70% of our respondents self-reporting lifetime experience of depression. Contrasting to existing evidence [34], however, overall rates were low for attention deficient/hyperactivity disorder (ADHD) and differences between groups were not statistically significant. When interpreting these data, it is important to stress the fact that both physical and mental health conditions were self-reported in this survey (i.e., not corroborated by clinical data). It is possible, therefore, that the higher rates of poor mental health and lower rates of ADHS identified were due to additional factors such as self-selected sample and/or social desirability bias.

We found high rates of adverse life experiences and trauma across the entire sample. Furthermore, whilst current ATS users were more likely to have grown up in care (where a child becomes the legal responsibility of a local authority) compared to either former or exposed non-users, we found no significant difference between groups in the rates of most other reported adverse childhood experiences. In part, this contrasts with both our cross-European qualitative interview findings and other research showing that exposure to and subsequent problematic use of stimulants and other illegal substances is more common amongst those with more negative life events (including parental substance misuse, physical or sexual abuse, and/or parental incarceration) [16,18,19,20]. Future research should seek to understand the relationship of specific events in the causal pathway of ATS use. However, significantly higher rates of adulthood experiences of familial estrangement (including homelessness and breakdown in living arrangements within parental/family home) amongst current and former ATS users (over four times and 2.5 times higher for current users compared to exposed non-users) were identified. This is consistent with current literature which shows prevalent stimulant and polysubstance use in individuals who experience homelessness or are in unstable housing [22,23]. The association between homelessness and ATS use seen in our study warrants further investigation to unpack the directionality of the relationship between homelessness and ATS.

### 4.1. Strengths and Limitations

A key strength of this study is its inclusion of and differentiation between current, former, and exposed non-ATS users. This allows key differences in the substance use profiles, self-reported mental and physical health, and lifetime adverse experiences of different user groups to be investigated and could help inform the development of more nuanced interventions and treatments in future. Additionally, the design and content of the survey questionnaire built directly on existing literature and the previous qualitative phase of the ATTUNE study, helping to generate an in-depth, contextualised understanding of a relatively under-researched group [17].

Non-probability quota-based sampling embedded within a wider European study was used for this study [24]. This approach means the direction of associations and causations cannot be assessed in our data. Furthermore, data collection took place in a single region of England, which may limit the external validity of our findings. Compared to other regions in England, the North East is less ethnically diverse and more socio-economically deprived [35]. Despite our best efforts, there was limited recruitment of individuals who self-identified as lesbian, gay, bisexual, transgender, queer or questioning, intersex, asexual, or those who are part of the community but are not captured within the previous sexual or gender identities (LGBTQIA+). This is problematic, given the higher prevalence of stimulant use within this community [36].

Despite the involvement of people with relevant lived experience in the survey design, feedback suggested it was overly lengthy and complex. This may have affected data quality and completeness. Furthermore, this could have led to self-selection bias in terms of the people who would be willing to participate. Additionally, to create a distinction between current and former users, a specific eligibility criterion was applied, whereby any individuals who had used ATS between 3 and 12 months prior to data collection were excluded. While this decision was taken so that the two populations could be could sufficiently differentiated for statistical comparison purposes, it could have resulted in the exclusion of potentially valuable data. Finally, the sample did not differentiate between dependent and occasional ATS use, and the high SDS scores suggest occasional ATS users may have been under-represented in our sample.

### 4.2. Implications

Nevertheless, our findings have several implications for policy and practice. First, these results lend further weight to calls for more concerted efforts to prevent early initiation of ATS use in adolescence. In general populations, evidence suggests that school-based interventions using a combination of social competence and social influence approaches may be most effective in this respect [37]. However, as shown here, certain contextual risk factors can increase the likelihood of both early initiation and continued ATS use, meaning targeted efforts are also needed: for example, to better support children growing up under local authority care.

Second, the high rates of mental ill-health reported by current and former ATS users here and in our previous qualitative studies [8,9,17] highlight the importance of substance use treatment that also incorporates evidence-based mental health support. Evidence remains sparse on effective approaches to treating co-occurring mental health conditions and substance use; however, existing guidelines suggest that effective care means first identifying the specific needs of individual users, and subsequently offering tailored and, where possible, integrated support and treatment based on their requirements [38,39].

Finally, given the additional social and economic challenges experienced by many ATS users, including homelessness, unemployment, low income, limited education, and offending history, our findings underline the importance of joined-up intervention programmes that recognise the wider structural factors that contribute to problematic substance use.

## 5. Conclusions

Early initiation or exposure to illicit substances, poor mental health, and experiencing certain adverse life events in childhood and adulthood are more common in individuals currently using ATS compared to former or non-ATS users. These findings suggest the need for both primary and secondary preventative interventions in adolescence to delay ATS initiation, alongside treatment programmes that can also address the mental health, social, and economic needs of problematic users. As the current user population continues to grow and age, the burden from ATS dependence will increase unless concerted efforts are made to improve treatment and prevention efforts [5].

## Figures and Tables

**Table 1:** Demographic information for survey participants by study groups.

		Current ATS Users (*n* = 137)	Former ATS Users (*n* = 174)	Exposed Non-ATS Users (*n* = 78)	Total (*n* = 389)	*p* Value
Gender	Female	50 (36.5%)	86 (49.4%)	47 (60.3%)	183 (47.4%)	0.007
	Male	85 (62.0%)	88 (50.6%)	30 (38.5%)	203 (52.6%)	
Age	Range (years)	18–61	19–68	18–64	18–68	0.024
	Mean (SD)	36.03 (9.6)	38.53 (9.2)	39.59 (12.8)	37.86 (10.2)	
Ethnicity	White British	128 (93.4%)	165 (94.8%)	72 (92.3%)	365 (93.8%)	0.328
Relationship status	Not currently in relationship	84 (61.3%)	101 (58.0%)	33 (42.3%)	218 (56.0%)	0.02
	Currently in relationship	53 (38.7%)	73 (42.0%)	45 (57.7%)	171 (44.0%)	
Annual net income	Less than GBP 18,000	123 (90.4%)	148 (85.1%)	46 (59.0%)	317 (81.7%)	<0.001
	GBP 18,000 to GBP 35,000	8 (5.9%)	18 (10.3%)	14 (17.9%)	40 (10.3%)	
	More than GBP 35,000	5 (3.7%)	8 (4.6%)	18 (23.1%)	31 (8.0%)	
Employment status	Any paid employment	14 (10.2%)	39 (22.4%)	42 (53.8%)	95 (24.4%)	<0.001
	Unemployed	35 (25.5%)	55 (31.6%)	7 (9.0%)	97 (24.9%)	
	Not in work due to sickness/disability	55 (40.1%)	47 (27.0%)	15 (19.2%)	117 (30.1%)	
	Not in work due to other reason	25 (18.2%)	32 (18.4%)	7 (9.0%)	64 (16.5%)	
	Full-time student	8 (5.8%)	1 (0.6%)	7 (9.0%)	16 (4.1%)	
Highest completed level of education	Basic level	55 (40.1%)	57 (32.8%)	7 (9.0%)	119 (30.6%)	<0.001
	Secondary level	36 (26.3%)	51 (29.3%)	15 (19.2%)	102 (26.2%)	
	Further education	43 (31.4%)	55 (31.6%)	43 (55.1%)	141 (36.2%)	
	Higher education	3 (2.2%)	11 (6.3%)	13 (16.7%)	27 (6.9%)	
Experience of prison	Ever been in prison	69 (51.1%)	63 (36.2%)	5 (6.4%)	137 (35.4%)	<0.001

**Table 2:** Differences in means and prevalence of licit and illicit substance use by study groups ^1^.

		Current ATS Users	Former ATS Users	Exposed Non-ATS Users	Total	*p* Value
Licit substance use		(*n* = 132)	(*n* = 170)	(*n* = 75)	(*n* = 377)	
AUDIT C-Score	Mean (SD)	5.29 (4.1)	5.36 (4.1)	5.19 (3.8)	5.30 (4.0)	0.992
	Score positive	74 (56.1%)	88 (51.8%)	41 (54.7%)	203 (53.8%)	0.749
	Score zero	23 (17.4%)	32 (18.8%)	9 (12%)	64 (17%)	0.417
Tobacco use	Never smoked	12 (8.8%)	25 (14.4%)	33 (42.3%)	70 (18.0%)	<0.001
	Current smoker	117 (85.4%)	134 (77%)	24 (30.8%)	275 (70.7%)	
	Former smoker	8 (5.8%)	15 (8.6%)	21 (26.9%)	44 (11.3%)	
Illicit substance use		(*n* = 137)	(*n* = 174)	(*n* = 78)	(*n* = 389)	
Age (years) first use or exposure	Mean (SD)	16.1 (4.7)	17.6 (4.5)	20.6 (8.3)	17.7 (5.7)	<0.001
SDS Score	Mean (SD)	5.9 (4.8)	4.1 (4.5)	N/A	4.9 (4.7)	<0.001
	Positive	79 (57.7%)	72 (41.4%)	N/A	151 (48.6%)	0.004
Any injecting drug use	Yes response	58 (42.3%)	30 (17.2%)	0 (0%)	88 (22.6%)	<0.001
Previous contact with specialist treatment	Yes response	105 (76.6%)	111 (63.8%)	14 (17.9%)	230 (59.1%)	<0.001

^1^ Data presented as number (percentage) of respondents unless otherwise indicated.

**Table 3:** Lifetime prevalence and mean age (years) of first use of specific ATS substances and other illicit substances ^1^.

		Current ATS Users	Former ATS Users	Exposed Non-ATS Users	Total	*p* Value
Specific ATS substances		(*n* = 137)	(*n* = 174)	NA	(*n* = 311)	
Amphetamine	Ever used	126 (92.0%)	158 (90.8%)	NA	284 (91.3%)	0.717
	Age of first use in years. Mean(S)	16.62 (5.0)	17.87 (4.8)	NA	17.32 (4.9)	0.001
MDMA	Ever used	124 (90.5%)	148 (85.1%)	NA	272 (87.5%)	0.149
	Age of first use in years. Mean(S)	17.6774 (5.1)	18.39 (4.7)	NA	18.07 (4.9)	0.013
Methamphetamine	Ever used	34 (24.8%)	19 (10.9%)	NA	53 (17%)	0.001
	Age of first use in years. Mean(S)	30.71 (10.1)	26.89 (10.6)	NA	29.34 (10.4)	0.15
Stimulant type NPS	Ever used	69 (50.4%)	44 (25.3%)	NA	113 (36.3%)	<0.001
	Age of first use in years. Mean(S)	30.04 (9.7)	28.89 (10.6)	NA	29.59 (10.0)	0.498
ATS medicine	Ever used	23 (16.8%)	28 (16.1%)	NA	51 (16.4%)	0.869
	Age of first use in years. Mean(S)	23.26 (8.3)	23.11 (8.4)	NA	23.18 (8.3)	0.977
Other illicit substances		(*n* = 137)	(*n* = 174)	(*n* = 78)	(*n* = 379)	
Cannabis	Ever used	127 (92.7%)	158 (90.8%)	41 (52.6%)	326 (83.8%)	<0.001
	Age of first use in years. Mean (SD)	14.48 (3.7)	16.27 (5.9)	18.63 (4.3)	15.87 (5.1)	<0.001
Opioids	Ever used	97 (70.8%)	85 (48.9%)	6 (7.7%)	188 (48.3%)	<0.001
	Age of first use in years. Mean (SD)	23.51 (8.3)	23.87 (7.3)	27.67 (7.4)	23.80 (7.8)	0.309
Cocaine/Crack	Ever used	128 (93.4%)	139 (79.9%)	10 (12.8%)	277 (71.2%)	<0.001
	Age of first use in years. Mean (SD)	20.94 (7.0)	22.06 (7.4)	27.9 (9.4)	21.75 (7.4)	0.01
Hallucinogens	Ever used	78 (56.9%)	92 (52.9%)	8 (10.3%)	178 (45.8%)	<0.001
	Age of first use in years. Mean (SD)	17.69 (6.8)	18.32 (5.0)	18.88 (3.3)	18.07 (4.8)	0.196

^1^ Data presented as number (percentage) of respondents unless otherwise indicated.

**Table 4:** Self-rated mental and physical health and self-reported lifetime prevalence of mental disorders by study group.

	Current ATS Users (*n* = 135)	Former ATS Users (*n* = 173)	Exposed Non-ATS Users (*n* = 78)	Total (*n* = 386)	*p* Value
Self-rated mental and physical health ^1^					
Current physical health (0–10)	5.59 (2.2)	5.71 (2.1)	6.95 (2.2)	5.92 (2.2)	<0.001
Current mental health (0–10)	4.87 (2.5)	5.41 (2.4)	6.55 (2.2)	5.45 (2.5)	<0.001
Self-rated mental disorders ^2^					
ADHD	7 (5.2%)	7 (4.0%)	1 (1.3%)	15 (3.9%)	0.356
Depression	102 (76.1%)	131 (75.3%)	48 (61.5%)	281 (72.8%)	0.043
Obsessive compulsive disorder	12 (9.0%)	17 (9.8%)	7 (9.0%)	36 (9.3%)	0.964
Eating disorder	17 (12.7%)	23 (13.2%)	5 (6.4%)	45 (11.7%)	0.268
Borderline personality disorder	23 (17.2%)	24 (13.8%)	5 (6.4%)	52 (13.5%)	0.085
Psychosis	42 (31.3%)	26 (14.9%)	3 (3.8%)	71 (18.4%)	0.000

^1^ Data presented as mean (SD) score. ^2^ Data presented as number (percentage) of respondents unless otherwise indicated.

**Table 5:** Prevalence of self-reported experience of adverse events in childhood and adulthood by study group ^1^.

	Current ATS Users (*n* = 135)	Former ATS Users (*n* = 173)	Exposed Non-ATS Users (*n* = 78)	Total (*n* = 386)	*p* Value
Childhood					
Experience of sexual assault until age 16 years	13 (9.6%)	16 (9.2%)	6 (7.7%)	35 (9.1%)	0.888
Experience of death of a parent in childhood	16 (11.9%)	19 (11.0%)	7 (9.0%)	42 (10.9%)	0.808
Experience of parental substance use	40 (29.6%)	43 (24.9%)	13 (16.7%)	96 (24.9%)	0.108
Experience of growing up in care	31 (23.0%)	19 (11.0%)	6 (7.7%)	56 (14.5%)	0.002
Adulthood					
Experience of sexual assault after age 16 years	25 (18.5%)	40 (23.1%)	8 (10.3%)	73 (18.9%)	0.054
Experience of homelessness after age 16	87 (64.4%)	88 (50.9%)	12 (15.4%)	187 (48.4%)	<0.001
Experience of breakdown in living arrangements within parental/family home	59 (43.7%)	63 (36.4%)	13 (16.7%)	135 (35%)	<0.001

^1^ Data presented as number (percentage) of respondents unless otherwise indicated.

## Data Availability

The data presented in this study are available on reasonable request from the corresponding author. The data are not publicly available due to ethical restrictions. The survey tool is available upon reasonable request from the corresponding author.
